# Improving knowledge, self-efficacy and collective efficacy regarding the Brazilian dietary guidelines in primary health care professionals: a community controlled trial

**DOI:** 10.1186/s12875-020-01245-3

**Published:** 2020-10-21

**Authors:** C. R. Tramontt, P. C. Jaime

**Affiliations:** 1 Nutrition in Public Health Postgraduate Program, School of Public Health, University of São Paulo, Av. Dr. Arnaldo 715, São Paulo, SP 01246-904 Brazil; 2 Department of Nutrition, School of Public Health, University of São Paulo, Av. Dr. Arnaldo 715, São Paulo, SP 01246-904 Brazil

**Keywords:** Interventional study, Capacity building, Food guides, Health personnel, Primary health care

## Abstract

**Background:**

Capacity-building of health professionals regarding to nutrition is a strategy for qualifying public health work to promote healthy diets in primary health care (PHC) services.

**Objective:**

To evaluate the effect of an intervention based on Brazilian Dietary Guidelines (BDG) on the knowledge, self-efficacy (SE) and collective efficacy (CE) of interprofessional teams working in PHC.

**Methods:**

It refers to a pre-post intervention study involving 24 health professionals divided into a control group (CG) and intervention (IG). The IG received a 16-h educational workshop on the BDG, guided by a validated protocol. Knowledge, SE and CE for using the BDG were assessed via a self-administered scale, ranging from 0 to 16 and 0 to 36 points, respectively; the scale was previously validated, completed before and after 2 months of the intervention. The effects of the intervention were estimated by paired t-test for intragroup comparisons over time.

**Results:**

The mean difference in the knowledge and SE scores of the IG pre- and post-intervention was 2.0 (CI 0.49–3.51) and 6.75 (CI 4.05–9.45) points, respectively. These results means the IG participants obtained 59 and 52.8% more points in knowledge and in SE in relation to CG, with significative difference (*p* = 0.007 and *p* <  0.00, respectively). There was no significant variation in the CE scores in both groups.

**Conclusions:**

Considering the results presented and due to the originality of the study in question, the educational workshop was effective in increasing the knowledge and SE of professionals working in PHC in using the Dietary Guidelines in their work routines. These findings can assist other research in developing nutrition interventions with interprofessional teams.

## Background

Healthy eating is an essential issue of health promotion and has gained increasing relevance in tackling chronic noncommunicable diseases worldwide [1]. The current epidemiological nutritional context in developing countries, where malnutrition and micronutrient deficiency coexist, and the emergence of an epidemic of overweight and obesity, underscore the importance of promoting healthy eating as a necessary component of the duties of professionals working in primary health care (PHC) [2–5].

Following the evolution in the organization of health work and in the recognition of the multidimensionality of factors that affect the diet, the promotion of healthy eating is increasingly being recognized as a collective effort, based on teamwork [3, 5]. Dietary Guidelines incorporate recommendations for a healthy diet and bring them closer to the regional realities of different countries, therefore, they are considered important tools to guide the performance of health professionals, especially at PHC services [6]. The new edition of the Brazilian Dietary Guidelines (BDG) brought an important update of the paradigm and recommendations on healthy eating. The BDG is internationally recognized for its innovation in considering emerging issues, such as sustainability, and for adopting a food classification based on the extent and purpose of industrial processing, recently assumed as a marker of global food quality [7, 8].

In Brazil, health has been recognized in the federal constitution as a right of citizens and a duty of the state since 1988. The Brazilian public health care system was established under the premises of universality, comprehensiveness and social participation [9] and the primary care is the entry point to the system. Considering this context, PHC professionals are key components in the dissemination of the Dietary Guidelines recommendations due to their access to and bonds with the population interacting with the various care levels of the health system [5, 6].

However, even though the potential impact of recommendations made by health professionals for changes in users’ behavior is recognized, little is incorporated into their practice, either due to the insufficient nutrition training offered to these professionals, the difficulty in addressing issues about food, and / or by possible barriers to implement changes in professional practice to guide individuals about food [10–12]. Considering these aspects, interventions that seek to increase knowledge and impact behavioral aspects such as self-efficacy and collective efficacy have been widely used in health education processes [12–14].

Self-efficacy and collective efficacy are constructs of the social cognitive theory, which refer to “an individual person’s perception of ability to perform a behavior” and “a group’s shared belief in its capability to organize and execute actions required to achieve goals” respectively [15]. Collective efficacy is considered an extension of the self-efficacy construct. In this sense, this study adopts these constructs believing that health professionals properly trained in nutrition improve their knowledge and their perception of self-efficacy and collective efficacy to counsel on health eating, either individually or as a team, improving the quality of care offered to the population.

Therefore, given the importance of these attributes in promoting healthy eating, this study aims to assess the effect of an educational intervention based on the Brazilian Dietary Guidelines on the knowledge, self-efficacy and collective efficacy of professionals working in PHC.

## Methods

### Design, setting and participants

To expand the coverage and problem-solving capacity of Brazilian primary health care system, were established Family Health Support Centres (NASFs, for its acronym in Portuguese), composed of multidisciplinary teams that work in an integrated manner with the PHC teams [16]. The NASFs operate according to the logic of interprofessional collaborative practice (ICP), which takes place when professionals from different areas provide services based on comprehensive health, involving patients and their families, caregivers and communities to provide the highest quality of care at all levels of the health care network [17].

In this context, the present study was a controlled community trial with a pre- and posttest design with professionals working in the NASFs of a medium-sized Brazilian municipality with approximately 400,000 inhabitants. All professionals registered and working in multidisciplinary NASF teams in the municipality were invited to voluntarily participate in the study. The municipality had four multidisciplinary NASF teams and each team was the reference team for approximately 8 to 10 basic health units, with the primary care system of the municipality covering approximately 53.7% of the population.

### Intervention: dietary guidelines for the Brazilian population workshop

The intervention protocol was developed intentionally to improve knowledge regarding to the Brazilian Dietary Guidelines (hereinafter referred to as the Guidelines), and to promote self-efficacy and collective-efficacy of health professionals teams to disseminate and translate the Guideline’s recommendations. The protocol was guided by three main theoretical references: the Guidelines, which are a technical evidence-based reference for the country in the field of food and nutrition; adult-learning theory, which is based on the critically reflective methodology developed by educator Paulo Freire [18], which in turn is based on the active participation of subjects, joint construction of knowledge and different teaching strategies; and the assumptions of ICP [17], linked to the PHC teamworks’ procedures, ICP occurs when different health professionals provide services based on a collaborative approach to care individuals and communities.

**Fig. 1:**
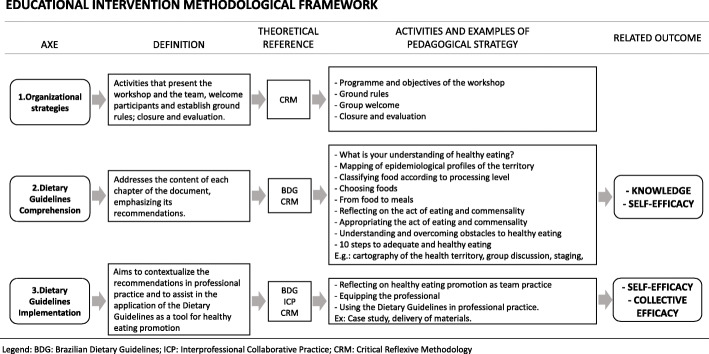
Educational intervention methodological framework for implementing the Dietary Guidelines for the Brazilian Population. Jundiaí, Brazil, 2019. Legend: BDG: Brazilian Dietary Guidelines; ICP: Interprofessional Collaborative Practice; CRM: Critical Reflexive Methodology

The educational intervention consisted of a workshop of 16 h of training divided into four four-hour modules. The activities were designed to allow the participants to rescue previous knowledge, analyse their work context reality and, from this, collectively construct concepts, reflect on their practice in a multiprofessional team and propose strategies for intervention at work using the Dietary Guidelines as the main technical reference for the activities. For that, the activities were developed and organized in three axes: (a) organizational strategies, referent to activities that present the workshop and the team, welcome participants and establish ground rules; closure and evaluation; (b) dietary guideline comprehension, addresses the content of each chapter of the document, emphasizing its recommendations; and (c) dietary guideline implementation, intended to contextualize the recommendations in professional practice and assist in the application of the guidelines as a tool for the promotion of healthy eating. The educational intervention methodological framework is described in Fig. 1.

The workshop was previously tested on five NASF teams from a municipality of similar size and context. With the purpose of testing its reproducibility in other teams in the country, the protocol was also validated by a panel of experts through content validation regarding its relevance, its clarity and the theoretical framework used for its development. The protocol and its validation are described in detail in a previous publication [19].

The health professionals were allocated nonrandomized into an intervention group (IG) and a control group (CG) according to the distribution of the teams in the municipality’s territory to minimize the cross contamination of information about the intervention between the groups.

**Fig. 2:**
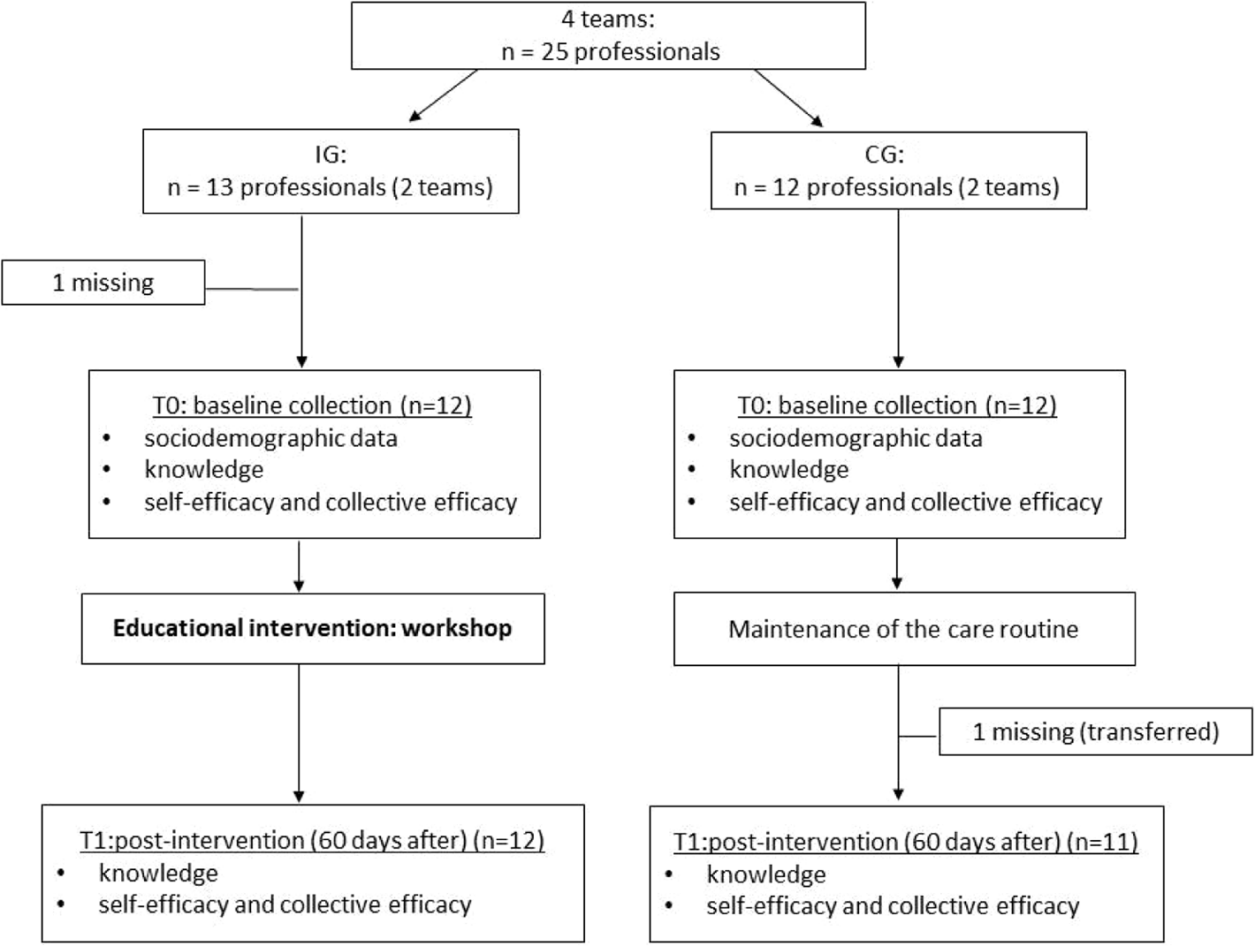
Flowchart of the educational intervention in multi-professional health teams for implementing the Dietary Guidelines for the Brazilian Population. Jundiaí, Brazil, 2019

The IG attended the workshop, which was led by facilitators previously trained by the team of responsible researchers and an observer who tracked the execution and adequacy of the intervention protocol. The workshop took place over 2 days during the working hours of the health teams. Participants in the CG did not change their routines during the intervention period. At the end of the study, the CG received the same training given to the IG. The flowchart for the study is shown in Fig. 2.

### Outcomes

The effect of the intervention was evaluated through the analysis of three outcomes: a) the health professionals’ *knowledge* about the content of the Guidelines, b) *self-efficacy* and *c)* *collective efficacy*, understood to be the confidence or belief in the ability of the individual or group, respectively, to develop and execute the actions necessary to achieve certain results or desired change, understood in this study as the confidence or belief in the ability to promote nutrition counseling based on the Guidelines.

Sociodemographic data were collected using self-administered questionnaires before the educational intervention. The outcomes of interest were measured using self-administered scales previously validated [20]. The knowledge scale consisted of 16 statements about the recommendations contained in the five chapters of the Guidelines, with three answer options (“true”, “false” and “I don’t know”). Each correct question gave participants a point, generating a knowledge score that ranged from 0 to 16 points. The scales that evaluated self-efficacy and collective efficacy were composed of 12 questions each with four-point responses on the Likert scale that assessed self-efficacy, ranging from 0 = not confident to 3 = very confident, and collective efficacy, ranging from 0 = false to 3 = very true. Each scale generated a final score ranging from 0 to 36 points. The score of knowledge, self-efficacy and collective efficacy was calculated using the average score of each group. The Table 1 shows samples of knowledge, self-efficacy and collective efficacy questions. The outcomes were evaluated on-site in both groups (IG and CG) before the intervention and 60 days after its completion by trained researchers who were not involved with the educational intervention. Understanding that the outcomes investigated in this study require time for professionals to be able to make connections with what was learned in the intervention and the work practice considering the dynamics of health services, it was assumed that the post-test would be better performed 60 days after the application of the intervention.

**Table 1:** Examples of knowledge, self-efficacy and collective efficacy questions used in each scale

Question	Score
**Knowledge**	
Potatoes, rice, beans, chestnuts and walnuts are foods that should be avoided to prevent weight gain.	1-True 2- False 3- Do not know
**Self-efficacy**	
I can advise health service users to combine foods in the form of healthy meals.	**0-**not confident **1-**a little confident **2-**confident **3-**very confident
**Collective efficacy**	
My team is able to advise health care users to combine foods in the form of healthy meals.	**0-** false **1-**sometimes true **2-**true **3-**very true

### Data analysis

All analyses were performed using Stata SE 14.1 (Stata Corp., College Station. Texas, USA). All questionnaires were double entered to ensure accuracy. All the normal distribution of the data was assessed by the Shapiro-Wilk test and a histogram. Differences in the sociodemographic characteristics at baseline for the IG and CG were analysed. For analyses between groups, the Fisher’s exact test was used to compare categorical data; Mann-Whitney test was used to analyse continuous variables with no equal distributions; and t-test was used for continuous data with equal distribution. A paired t-test was used for intragroup comparison at T0 versus T1. Statistical significance was accepted at the level of *p* <  0.05.

This study was approved by the research ethics committee of the University of São Paulo School of Public Health (Faculdade de Saúde Pública da Universidade de São Paulo) and by the Municipal Health Department, and the participants voluntarily signed an informed consent form.

## Results

The characteristics of the study groups are presented in Table 2. The IG and CG did not differ at baseline with regard to the sociodemographic characteristics, professional categories that composed the teams, length of professional training, time working in the team or with regard to the knowledge, self-efficacy and collective efficacy scores. Eight professionals in each group reported already having knowledge of the guidelines.

**Table 2:** Sociodemographic characterization and distribution of outcome scores of the groups at baseline (*N* = 24). Jundiaí, Brazil, 2019

	Group		
	Control n (%)	Intervention n (%)	Significance test
**Sex**			
M	4 (33.3)	3 (25)	
F	8 (66.7)	9 (75)	0.203*
**Occupation**			
Social worker	2 (16.7)	2 (16.7)	
Physical educator	2 (16.7)	3 (25)	
Physical therapist	2 (16.7)	2 (16.7)	
Nutritionist	2 (16.7)	1 (8.3)	
Psychologist	2 (16.7)	2 (16.7)	
Occupational therapist	2 (16.7)	2 (16.7)	0.533*
**Did you have prior knowledge of the Dietary Guidelines?**			
Yes	8 (66.7)	8 (66.7)	
No	4 (33.3)	4 (33.3)	1.000*
	**Mean (SD)**	**Mean (SD)**	
**Age**	42.08 (11.18)	37.36 (8.3)	0.3548**
**Length of professional training (years)**	15.92 (12.27)	10 (7. 27)	0.3875**
**Time working at the FHSC (months)**	24. 9 (11.82)	21.08 (11.85)	0.416**
	**Mean (CI)**	**Mean (CI)**	
**Knowledge score**	12.25 (10.67 to 13.83)	10.83 (9.43 to 12.24)	0.1546^a^
**Self-efficacy score**	18.25 (1.69 to 5.61)	16.08 (24.33 to 31.83)	0.3748^a^
**Collective efficacy score**	23.58 (19.28 to 27.89)	25.42 (21.73 to 29.11)	0.4841^a^

*Fisher’s exact test: p < 0.05

**Mann-Whitney test *p* < 0.05

^a^Student’s t-test

In the analysis of the variation over time (T1-T0), the outcomes with the greatest variations in the IG were self-efficacy, with a mean score difference of 6.75 points (*p* < 0.001), followed by knowledge, with a mean score difference of 2.0 points (*p* = 0.007), which means an increase of 59 and 52.8% on knowledge and perception of SE, respectively, in those professionals who participated in the educational intervention. There was no significative variation on CE scores over time. For the CG, there were no significant variations in the mean scores of the outcomes before and after the intervention. (Table 3).

**Table 3:** Effect of the intervention on knowledge, self-efficacy and collective efficacy (*N* = 24). Jundiaí, Brazil, 2019

	Intervention group (***n*** = 12) mean (95% CI)				Control group (***n*** = 11) mean (95% CI)			
	T_**0**_	T_**1**_	Δ	p	T_**0**_	T_**1**_	Δ	p
**Knowledge**	10.83 (9.43 to 12.24)	12.83 (10.72 to 14.95)	2.0 (0.49 to 3.51)	0.007	12.25(10.67 to 13.83)	13.64 (11.88 to 15.39)	0.82 (−0.34 to 1.97)	0.073
**Self-efficacy**	16.08 (12.33 to 19.83)	22.83 (19.63 to 26.04)	6.75 (4.05 to 9.45)	< 0.001	18.91 (15.14 to 22.68)	22.09 (16.29 to 27.89)	3.18 (−0.74 to 7.11)	0.051
**Collective efficacy**	25.42 (21.73 to 29.11)	25.25 (21.64 to 28.86)	−0.17 (−2.83 to 2.49)	0.553	23.09 (18.47 to 27.71)	20.82 (15.35 to 26.29)	−2.27 (−6.59 to 2.04)	0.866

## Discussion

The present study assessed an educational intervention undertaken with professionals of Family Health Support Centres seeking to improve the knowledge, self-efficacy and collective efficacy to promote health eating based on Brazilian Dietary Guidelines. The results were positive in terms of improving the professionals’ knowledge and self-efficacy regarding the current recommendations for health eating in the IG compared over time.

The change in the level of knowledge is commonly the most prevalent outcome of interest in studies that carry out some type of training in nutrition with health professionals, followed by changes in attitudes, self-reported practices, self-efficacy, confidence and feedback [21, 22].

In most cases, knowledge is positively impacted by interventions; however, some methodological differences can be found when compared to this study. The absence of comparison with CG is the main point of divergence, followed by aspect referred to the knowledge investigation questionnaires, which for the most part have not been validated for use in the study, raising questions about the validity of the answers found, and confirm the reliability of the results presented by this study [21, 22]. In addition, another relevant factor is the time after the intervention within which knowledge is measured. Knowledge is usually measured within a short period of time to minimize participants’ memory bias [21]. Knowledge is usually lost over time if not put into practice or resumed and must be relearned throughout a continuous education process [23]. This study, however, demonstrated that the participants obtained a relevant gain of knowledge even after 2 months of the educational intervention. This may mean that the participants, in addition to increasing knowledge, were able to retain the knowledge acquired in the medium term – 60 days after the intervention. A short- and long-term evaluation would allow confirmation and assessment of the permanence of the effect over time.

The perception of self-efficacy increased significantly within the time variation in this population. Few studies have found positive results for self-efficacy, either alone or in combination with knowledge [21]. It is a consensus in the literature that professionals who, in addition to being better trained, feel confident in performing certain actions are more likely to rethink their practices and change their attitudes [24, 25].

According to the SCT, self-efficacy is mutually influenced by individuals’ behavioral, environmental and personal factors [15], characteristics which do not depend exclusively on the educational intervention delivered. In this study, the format of the educational intervention – which made use of active teaching methods that imparted meaningful learning to the participants and which sought to develop skills and not only knowledge –, may have convincing participants about the relevance of the theme, since individuals seemed to feel more confident to advise about healthy eating based on the Dietary Guidelines. According to Mogre et al., teaching methods play a decisive role in the positive impact of nutritional interventions [21].

Another important point to be discussed is that part of the effect of the intervention on knowledge and SE, can be attributed to the educational tool itself - the Brazilian Dietary Guidelines. Its expanded approach on the dimensions that involve food, meals and ways of eating, considering biological, cultural, economic and environmental aspects, may have come closer to the competences perceived by these health professionals. NASFs who received the educational intervention seemed to appropriate of the content delivered and identified that advice on healthy eating is part of its duties, considering the logic of interprofessional collaborative practice.

No effect of the educational intervention was observed on the collective efficacy of the health professionals comparing post and pre-intervention time. In fact, both groups already had high CE at the beginning of the study, which made it difficult to demonstrate an effect on this outcome. This result may be related to factors external to the intervention that are associated with team-work dynamics. The fact that the professionals trusted in the capabilities of their teams to advise on healthy eating, perhaps means that they performed satisfactory teamwork. Therefore, the efficient interprofessional collaborative practice performed by these teams, highly supported by the local government in the context of this study, may have contributed to their high perception of CE [26, 27]. This result reinforces the importance of the workforce qualification processes being supported by a policy of continuing health education, which strengthens the premises of interprofessional collaborative practice at PHC [27, 28].

Previous studies have noted that achieving effective ICP impacts is complex and requires interventions that focus on improving communication, stimulating trust among team members and investing in intersectorally coordinated actions such that the health care processes become more durable [17, 29, 30]. Work settings and organizational barriers are fundamental to understanding the feasibility and effectiveness of interventions that are applied under real working conditions [28, 30]. In this sense, to promote healthy eating guidelines, further research should be conducted to stimulate the development of competencies, self-efficacy and specific skills for collaborative teamwork, considering that these multidisciplinary teams have been successful models to health systems organization [31].

Although the participants were not randomly allocated to the intervention or control groups in the present study, the differences between the groups at baseline were not significant, and therefore, should not have interfered on the results presented in this study.

One additional factor deserving of consideration is the possible induction effect resulting from the completion of questionnaires by the participants prior to the intervention. This action might have favourably influenced the results of the second assessment by raising the professionals’ awareness regarding to the investigated topics, thereby leading them to seek information, discuss the topics more frequently in their routines, or both, typical of studies applied under real service conditions. However, because significative increased on outcomes investigated based on the second questionnaire was not observed in the control group, it may be inferred that the results were due to the educational intervention.

The validity of the results is also supported by the procedure used to assess the between-group differences regarding the knowledge and perception of self-efficacy of health professionals. Traditionally, assessment of this outcomes is based on professionals self-reports [21, 32]. The scales used to assess the outcomes in this study were previously validated by a rigorous five-step process: content validation with panel of experts, face validation with potential users, online reevaluation by health professionals and experts, online application with PHC professionals working all over Brazil’s macro-regions and confirmatory factor analysis to investigate construct validity [33]. The use of these scales to assess the knowledge of health professionals about the dietary guidelines, as well as their self-efficacy and collective efficacy preceptions to guide healthy eating, guarantees more reliability to the results found.

The small sample size in this study is a limiting factor for more robust analyzes typical of clinical trials, which hinders the reproducibility and extrapolation of the results. However, the nature of the intervention performed in a real service context, and the positive effect observed on the IG, are strengths of this study. Most studies on educational intervention in nutrition focus only on doctors, nurses or graduate students, and aim to implement clinical protocols or recommendations focused on a determined aspect of nutrition, such as professional training on breastfeeding [21, 34–37]. Few studies carry out training of health teams with such diversity of professional categories as presented in this study. It is therefore believed that the population of this study is a representative sample of professional diversity in the Brazilian PHC system. In light of methodologies commonly used in nutrition intervention studies, we also highlight that the methodological rigor sought in all steps of this study is scarce to find in literature.

The applicability of the intervention protocol that was developed and tested in the present study is also highlighted. It was published by the Brazilian Ministry of Health as an instructional manual entitled “Implementing the Dietary Guidelines for the Brazilian Population in Teams Working in Primary Care (*Implementando o Guia Alimentar para a População Brasileira em equipes que atuam na Atenção Básica*) [38]. Brazil has a recognized history of implementing governmental strategies to promote healthy eating practices [39–41], and this represents an established initiative. The publication of this material opens up possibilities for the intervention to become a national strategy for training professionals, contributing to the implementation of the Dietary Guidelines in health teams throughout the country. Its country-wide dissemination will allow further evaluation to be performed using larger and more representative samples to compare the results presented in this study.

Since the professionals who participated in the training about Brazilian Dietary Guidelines increased their knowledge and felt more confident to advise on food, the findings of this study point to the need for investments in capacity building for health professionals to deal with nutrition issues, in view of the emergence of the current epidemiological scenario. There is a need for nutrition to be recognized as an interdisciplinary field and to be considered from professional training, including it in higher education curricula. In addition to academic training, it is important to consider that continuing professional education processes must be carried out, including training in nutrition, in order to improve multiprofessional teams’ practice.

We recognize the need for future studies to determine whether the educational intervention influenced the nutritional counseling behavior of professionals in the real context of in-service practice.

## Conclusions

The educational intervention demonstrated to be effective in increasing knowledge about the Dietary Guidelines and improving the perception of self-efficacy to advise on healthy eating in health professionals. The results presented in this study reinforce the importance of training PHC health professionals on nutrition. In addition, it legitimizes the importance of investing in continuing education processes in multiprofessional health teams for the implementation of official dietary guidelines.

## Data Availability

The datasets used and/or analysed during the current study are available from the corresponding author on reasonable request.

## Abbreviations

BDG: Brazilian dietary guidelines
CG: Control group
CE: Collective efficacy
ICP: Interprofessional collaborative practice
IG: Interventional group
NASF: Family Health Support Centres (Núcleo de Apoio a Saúde da Familia – NASF for its acronym in Portuguese)
PHC: Primary Health Care
SE: Self efficacy
